# A comparative study of mothers of infants hospitalized in an open ward neonatal intensive care unit and a combined pod and single-family room design

**DOI:** 10.1186/s12887-020-1929-1

**Published:** 2020-01-29

**Authors:** Nancy Feeley, Stephanie Robins, Christine Genest, Robyn Stremler, Phyllis Zelkowitz, Lyne Charbonneau

**Affiliations:** 1 Centre for Nursing Research, Jewish General Hospital, 3755 Côte-Ste-Catherine Rd, B- 621, Montréal, Québec H3T 1E2 Canada; 2 Ingram School of Nursing, McGill University, Montréal, Canada; 3 Institute of Community and Family Psychiatry, Jewish General Hospital, 4333 Côte Ste-Catherine Road, Montreal, Quebec H3T 1E4 Canada; 4 Faculty of Nursing, University of Montreal, 2375 Côte Ste-Catherine Road, Montréal, Québec H3T 1A8 Canada; 5 Lawrence S. Bloomberg Faculty of Nursing, University of Toronto, 155 College Street, Suite 130, Toronto, Ontario M5T 1P8 Canada; 6 Department of Psychiatry, Jewish General Hospital Senior Investigator, Lady Davis Institute for Medical Research, 4333 Côte Ste-Catherine Road, Montréal, Québec H3T 1E4 Canada; 7 Neonatology, Jewish General Hospital, 3755 Côte-Ste-Catherine Rd, Montréal, Québec H3T 1E2 Canada

**Keywords:** Neonatal intensive care, Design, Mothers, Stress, Depression, Family-centered care, Support from nurses, Breastfeeding self-efficacy

## Abstract

**Background:**

The well-being of mothers of infants requiring Neonatal Intensive Care Unit (NICU) hospitalization may be affected by the architectural design of the unit. A few recent studies suggest there may be some drawbacks of single-family rooms (SFRs) for infants and their mothers, such as isolation of mothers and reduced exposure to auditory stimulation for infants.

**Purpose:**

To compare NICU-stress, symptoms of depression, perceptions of nurse-parent support and family-centered care, sleep disturbances, breastfeeding self-efficacy and readiness for discharge in mothers of infants cared for in an open ward (OW) to those cared for in a unit that includes both pods and SFRs.

**Methods:**

A pre-post quasi-experimental study was conducted in a Canadian level 3 unit before and after transitioning to a new unit of 6-bed pods and SFRs. OW data were collected in 2014 and pod/SFR data 1 year after the transition in 2017 to 2018. Mothers of infants hospitalized for at least 2 weeks completed questionnaires about stress, depressive symptoms, support, family-centered care, and sleep disturbances. In the week prior to discharge, they responded to breastfeeding self-efficacy and readiness for discharge questionnaires. They described their presence in the NICU at enrollment and again prior to discharge.

**Results:**

Pod/SFR mothers reported significantly less NICU-stress compared to OW mothers. OW mothers had greater sights and sounds stress and felt more restricted in their parental role. Pod/SFR mothers reported greater respect from staff. Controlling for maternal education, pod/SFR mothers perceived their infant’s readiness for discharge to be greater than OW mothers. There were no significant differences between groups in depressive symptoms, nurse-parent support, sleep disturbances, and breastfeeding self-efficacy.

At enrollment and again in the weeks preceding discharge, pod/SFR mothers were present significantly more hours per week than OW mothers, controlling for maternal education.

**Conclusions:**

Further study of small pods is indicated as these units may be less stressful for parents, and enhance family-centered care, as well as maternal presence, compared to OWs.

## Background

The trend in Neonatal Intensive Care Unit (NICU) architectural design has been to build single family rooms (SFRs) or replace existing open ward units (OW) with single family rooms (SFRs) [1]. OWs house all infants and their families in one room while SFRs afford a private room for each family and their infant or infants. Another room design option is pods with a cluster of 4 to 6 infants cared for in one space. Evidence is needed to guide decision-making for those planning to renovate or build. A systematic review and meta-analysis of 13 studies appearing between 2004 and 2018 found that SFR NICUs have a lower incidence of infant sepsis and higher rates of exclusive breastfeeding at discharge compared to OWs. In four of six studies, parental presence, participation in infant care and skin to skin contact were also greater in SFRs. However, no differences were found in infant major morbidities, length of hospital stay, and developmental outcomes [2]. Many previous studies were limited by lack of reliable and valid measures of the constructs of interest and small sample size [2, 3]. Some well-designed studies have reported unexpected negative outcomes for SFR infants and their parents, such as poorer infant language development and greater maternal stress [4, 5], perhaps due to isolation, raising concerns about SFRs. This has led some design experts to suggest including both SFRs and small pods, and tailoring room assignment to the infants’ and families’ needs [6].

Parents of infants in NICUs can spend many weeks and even months in the unit. Architectural design may also affect parents, and its impact on their well-being requires further research [7]. Decades of research provide evidence that elements of the NICU environment and experience are stressful for mothers. Mothers of NICU infants have more depressive symptoms up to 1 year postpartum; and rates of clinical depression are as high as 40% in the first 3 months [8]. Rates of depression vary greatly across units, prompting speculation that design may play a role [9, 10]. Few studies examine mothers’ NICU-stress (that is stress arising from the NICU environment and hospitalization), and depressive symptoms in different design environments, and findings thus far are conflicting. An American study found that NICU-stress levels, not depressive symptoms, were higher among mothers in an OW compared to SFR mothers [11]. Yet in another study, SFR mothers had greater NICU-stress at discharge compared to OW mothers [5], but no differences were found in depressive symptoms, anxiety, or confidence. This unexpected finding led to speculation that in SFR units, nurses may be less visible or available to mothers leading them to feel more responsible for infant care.

A survey of parents whose infant was hospitalized in an OW and subsequently moved to a new SFR unit found that parents felt better supported by nurses in the SFR unit [12]. However, to our knowledge no design studies have investigated with reliable and valid measures mother’s perceptions of nurse support. The SFR design is also thought to optimize the provision of family-centered care as it provides facilities and space for parents to comfortably remain at the bedside and care for their infant [13]. One previous study has explored perceptions of family-centered care and found that compared to mothers of infants hospitalized in an OW, mothers in an SFR unit perceived care as more family-centered [14].

Increasing evidence points to the importance of parent’s presence at the bedside or involvement in infant care for the development of newborns hospitalized in NICUs. Recent studies have found that presence or involvement is associated with better infant reflex development at term age, better cognitive and language development at 18 months, and better motor development at 4 to 5 years of age [11, 15]. Moreover, there appear to be benefits for institutions. NICU hospitalization is costly, and every day of maternal involvement has been associated with a 4.3 day decrease in the length of infants’ hospitalization [11].

Mothers with an infant in the NICU report sleep disturbances such as night waking even though they slept at home, not in hospital [16]. A systematic review revealed that parents of preterm infants obtain less than the recommended hours of sleep both during the hospitalization and following discharge [17]. Moreover, their sleep quality measured subjectively or objectively is poor. Poor sleep is linked to adverse health outcomes, thus determining the influence of NICU design on mothers’ sleep is essential but has yet to be examined.

Among infants who require NICU care, breast milk feeding is associated with better cognitive development, fewer re-hospitalizations, greater brain volume and white matter, and lower rates of sepsis, retinopathy of prematurity, and necrotizing enterocolitis [18–20]. The evidence to date shows that breastfeeding rates at discharge are higher in SFR units [2]. Privacy, less noise and more space may facilitate milk expression, skin to skin contact and feeding at the breast. Breastfeeding self-efficacy, or how capable a mother feels about her ability to breastfeed, is a predictor of breastfeeding duration in mothers of NICU infants [21] and may be higher in SFR or pod designs for these reasons. To our knowledge, no previous design studies have examined mothers’ breastfeeding self-efficacy.

An infant’s discharge from the NICU is a stressful event for parents. They may not feel ready to assume full responsibility of the care of their infants after weeks or months in hospital. In an environment where mothers feel supported by nursing staff and have privacy to learn to care for their infant, they may be better able to learn and then perceive they are ready to assume caring for their infant upon discharge home. Mother’s readiness for their infant’s discharge from units of differing designs warrants exploration.

In 2016 the NICU of a tertiary care hospital moved from an OW in an old wing of the hospital to a newly constructed pod/SFR unit in a newly constructed critical care wing. This event provided an opportunity to investigate the well-being and presence of mothers in a unit design not typically studied. We hypothesized that mothers in the pod/SFR unit would have lower NICU-stress compared to mothers whose newborn was cared for in the former OW. Also, we expected that pod/SFR mothers would report fewer symptoms of depression, greater family-centered care, perceive greater support from nurses, and fewer sleep disturbances compared to a cohort of mothers whose infants were cared for in the OW. Lastly, we proposed that their breastfeeding self-efficacy and readiness for discharge would be greater than OW mothers.

## Methods

### Design

A pre-post quasi-experimental study was conducted. Following research ethics approval by the Research Ethics Office of the Jewish General Hospital (Federal Assurance Number 0796), data were collected from mothers who provided written informed consent in the OW unit from February to December 2014. The pod/SFR was scheduled to open mid-2015, however this was delayed until January 2016. To allow time for the transition and adjustment of the staff to the new unit, post-occupancy data collection started 1 year after the transition beginning May 2017 and were collected to May 2018.

### Participants and setting

Mothers were included if their infant was hospitalized in the NICU for at least 2 weeks and was considered stable by the attending neonatologist at the time of recruitment; they were able to read English or French and provide informed consent; and they were living less than 1 hour from hospital during the hospitalization. They were not included if: they would not be caring for the infant after discharge (e.g., foster placement); the infant had a major congenital anomaly, or sensory handicap; or they had given birth to multiples.

The primary outcome in this study was maternal NICU-stress measured with the Parental Stress Scale: Neonatal Intensive Care Unit (PSS: NICU). Based on a previous study [15], the standard deviation for the PSS: NICU score was expected to be around 1.2 and a difference of 0.75 is considered clinically significant. A sample size of 56 mothers in the OW and 56 mothers in the pod/SFR unit would provide 80% power to detect such a difference. To allow for attrition, 70 mothers were enrolled in the OW and 80 in the pod/SFR unit.

**Fig. 1:**
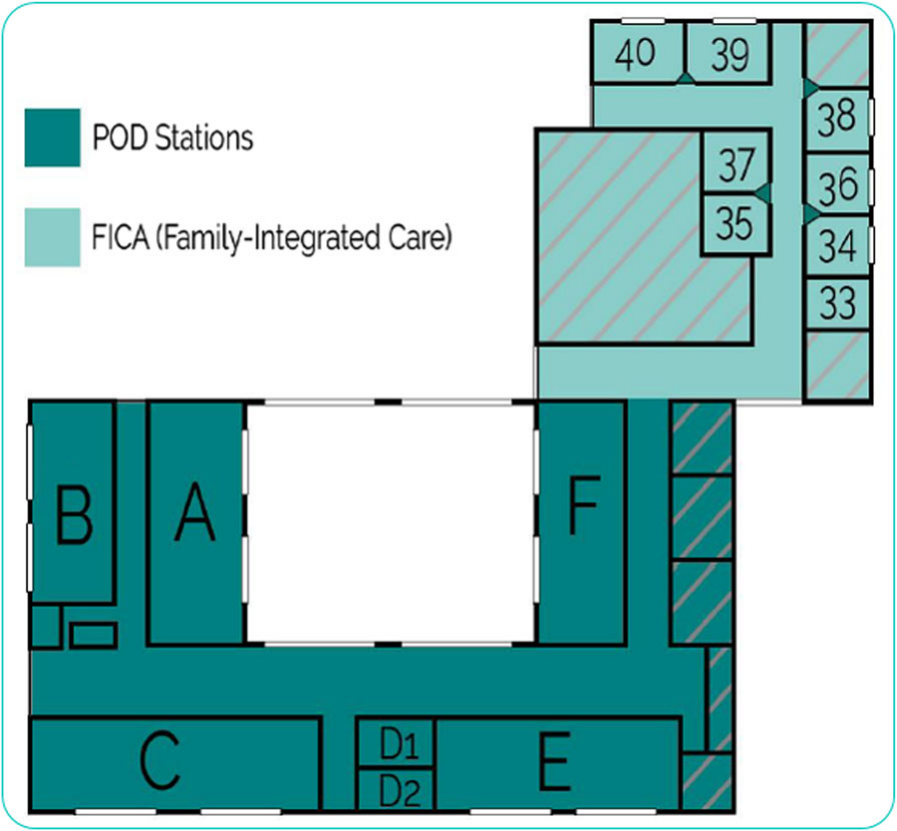
Pod/SFR unit design

The study was conducted at a Canadian level 3 NICU that transitioned from a 34-bed OW design to a 40-bed combination design, consisting of three pods of six beds for level 3 intensive care (1:2 nurse-patient ratio), two pods similar in size of six beds for level 2 care (1:3 ratio), and 10 SFRs for level 1 care (1:4 ratio) (Fig. 1: Pod/SFR unit design). In this new unit, all infants are admitted to a pod and, once their condition permits, moved to a SFR when they require step-down care. Therefore, in contrast to most previous NICU design studies our pod/SFR participants experienced two types of design: their infant was admitted to a 6-bed pod and moved to a SFR for step-down care prior to discharge.

The former OW was a 34-bed level-3 unit of 400-m^2^ in one space with around 550 admissions per year, including about 115 infants born very low birthweight (less than 1500 g). Six neonatologists and 94 nurses were on staff. There was one room designated for mothers to express breast milk, and a parent room with one sofa bed located down a hallway outside the unit for over-night stays. Parents could place a chair at their infant’s bedside. The OW had florescent lighting and windows on only one of the four walls.

The current 1125-m^2^ pod/SFR unit is situated in a newly constructed wing, and has new ventilators, monitors, and robotic arms. There are 7 neonatologists and 91 nurses on staff who work in both the pods and SFR areas. There are two rooms dedicated for parents to sleep overnight on a full-size bed (these must be reserved), and each SFR includes a parent lounger chair that does not lay fully flat but can also be used for overnight stays. In the new unit there is one shared parent shower room, and a dining area with kitchen facilities just outside the entrance is designated for their use. In the new unit, the lighting is indirect florescent; and in all pods, there are large windows with blinds for light control.

Both pre- and post-move parents had access to the unit 24-h per day and were invited to attend weekly parent meetings on topics of interest. Although several staff nurses were trained lactation consultants, there was no dedicated position for lactation support in the former OW nor the current pod/SFR.

### Measures

All the major study dependent variables were assessed with reliable and valid measures, most previously used in studies of mothers of NICU infants. The revised Parental Stress Scale: Neonatal Intensive Care Unit (PSS: NICU) [22 ;Miles MS. Parental stress scale: neonatal intensive care unit. Unpublished Questionnaire. 2002] assessed the primary dependent variable: maternal stress related to the experience of their infants’ hospitalization. This self-report measure is the gold standard for measuring parent stress in this context and taps stress related to NICU sights and sounds, feeling restricted in their role as caregiver, and the infant’s behavior and appearance. Higher scores reflect greater stress. Internal consistency is excellent, and there is evidence of construct validity [22, 24].

The Edinburgh Postnatal Depression Scale (EPDS) [24] is widely used to assess perinatal depressive symptomatology with demonstrated high internal consistency and validity for detecting major depression in the perinatal period. We used a cut-off score of 12 or more to indicate possible clinical depression as this score has optimal sensitivity for a diagnosis of major depression [25]. Mothers’ perceptions of staffs’ ability to provide the infant and family with family-centered care during hospitalization was measured with the Family Centered Care Questionnaire - Parents (FCCQ) [26]. The FCCQ examines respect, collaboration and support; and both validity and reliability are established.

The Nurse Parent Support Tool (NPST) is designed to measure parents’ perceptions of nursing support during their child’s hospitalization [27], and higher scores indicate higher support. Internal consistency is high and the NPST discriminates among parents with different levels of support [27]. The General Sleep Disturbance Scale (GSDS) [28] assesses subjective sleep disturbance. The GSDS has well-established validity and reliability in parents of newborns, and a score above 42 indicates sleep disturbances.

The Breastfeeding Self-Efficacy Scale - Short Form (BSES-SF) measures mothers’ breastfeeding confidence [29]. Higher scores indicate greater self-efficacy. Reliability estimates indicate excellent internal consistency, and it has been validated with NICU mothers [30]. The Readiness for Hospital Discharge Scale (RHDS) assessed mothers’ perception of their preparedness for leaving the NICU. Items measure the parents’ preparedness (parent subscale), as well as their perception of how ready their infant is to go home (child subscale) [31]. Predictive validity has been established [31].

At enrollment, data on maternal age, education, marital and employment status, parity, country of birth and other demographic characteristics were also collected with a background questionnaire. At enrollment and again in the week prior to their infant’s discharge, participants reported the number of hours and timing of their presence in the NICU each day for the previous 7-day period.

### Data collection procedures

Following research ethics approval, research staff approached mothers who met inclusion criteria and agreed to learn about the study. Those who subsequently provided written informed consent were issued an on-line secure website address at enrollment to access and complete questionnaires about stress, depressive symptoms, support, family-centered care, and sleep disturbances. They also responded to the socio-demographic questionnaire and described their presence. At enrollment, mothers took 20 to 30 minutes to complete the questionnaires. Paper copies of all questionnaires were available for those who preferred and completed in the NICU. At discharge, data about their infant’s medical condition and birth were extracted from the hospital medical record by research staff.

In the week prior to their infant’s anticipated discharge from hospital, mothers completed the breastfeeding self-efficacy and readiness for discharge questionnaires and again responded to the question about their presence in the NICU. For both groups, retention was very good: 80% of mothers completed data collection at discharge in the OW group and 77% in the pod/SFR group.

### Data analyses

Range and logic checks were conducted for all study variables. To describe the sample demographic and clinical characteristics, percentages were computed for categorical variables and means and standard deviations for continuous variables. To compare group characteristics, *t*-test and Wilcoxon rank sum test were used for continuous variables. Chi square and Fisher’s exact test were used for categorical variables.

T-test and Wilcoxon rank sum test were conducted to compare the groups’ unadjusted means for the maternal dependent variables, and analysis of covariance (ANCOVA) was used to adjust for covariates. All tests performed were two-sided and a *p* value of less than or equal to 0.05 was considered significant.

## Results

The demographic and clinical characteristics of the two groups of mothers and their infants are reported in Tables 1 and 2. The two groups were comparable with respect to infant and maternal characteristics with one exception: a significantly greater proportion of pod/SFR mothers had university education compared to OW mothers. Thus, one-way ANCOVAs were conducted to determine if there was a statistically significant difference between groups on the maternal dependent variables controlling for maternal education. Given there were very few differences in our findings, we report the unadjusted means in Table 3 but describe below any differences when adjusting for education.

**Table 1:** Characteristics of Mothers in the Open Ward (*N* = 70) and Pod-SFR Units (*N* = 80)

Characteristic	Open Ward		Pod-SFR		Test of Comparison (*p* values)
	*M*	*SD*	*M*	*SD*	
Age (years)	31.97	5.77	33.05	5.43	.24
Years living with partner	6.13	4.72	5.98	4.92	.69
Time to travel to hospital (minutes)	39.81	22.80	36.20	19.65	.40
	n	%	n	%	
Education: High School or Junior college	42	60.00	35	43.75	.04
Canadian citizen	55	78.57	71	88.75	.09
First born child	35	50.00	52	65.00	.08
Marital status: Partnered	64	91.43	75	93.75	.59

*Notes:* Statistical tests of comparison: Chi-square for categorical variables; Wilcoxon test for continuous variables except age; t-test for age

**Table 2:** Infant Characteristics in the Open Ward (*N* = 70) and Pod-SFR Units (*N* = 80)

	Open Ward		Pod-SFR		Test of Comparison (*p* values)
Characteristic	*M*	*SD*	*M*	*SD*	
Gestational age at birth (weeks)	30.27	3.20	30.04	3.32	.67
Birth weight (grams)	1401.36	521.79	1422.08	586.98	.82
Days hospitalized	54.27	30.48	55.50	32.97	.99
Days on respiratory support	27.83	29.49	29.99	30.01	.57
	*n*	%	*n*	%	
Gender: Female	36	51.43	35	43.75	.35
Intraventricular hemorrhage: Grade 2 or less	66	94.29	78	97.50	.42
Retinopathy of prematurity: Negative	68	97.14	78	97.50	1.00
Periventricular leukomalacia: Negative	69	98.57	80	100.00	.47

*Notes.* Statistical tests of comparison: t-test for gestational age and birth weight; Wilcoxon test for the other continuous variables. Chi-square for gender; Fisher’s exact test for the other categorical variables

**Table 3:** Comparison of Mothers in the Open Ward and the Pod-SFR Units

	Open Ward		Pod-SFR		Test of Comparison (*p* values)
	*M*	*SD*	*M*	*SD*	
At enrolment					
NICU Stress (PSS: NICU)					
Sights & sounds	2.75	0.83	2.47	0.71	.05 (.008)^1^
Infant behavior & appearance	3.03	1.01	2.87	0.86	.30
Parental role restriction	3.36	0.88	2.94	0.77	.002
Total score PSS: NICU	3.10	0.76	2.83	0.70	.03
Symptoms of depression (EPDS)	11.11	5.71	10.76	5.86	.72
Nurse-Parent Support (NPST)	4.07	0.65	4.27	0.59	.07
Family-Centered Care (FCCQ)					
Respect	3.28	0.40	3.43	0.43	.04
Collaboration	3.00	0.51	3.12	0.43	.19
Support	2.92	0.56	3.04	0.56	.26
Total score FCCQ	3.07	0.41	3.19	0.39	.06
Sleep disturbances (GSDS)	57.70	19.66	54.10	22.17	.33
Presence per week at enrolment (hours)	37.71	17.42	42.37	26.63	.72 (.02)^1^
In weeks preceding discharge					
Breastfeeding Self-efficacy (BSES)	50.04	13.70	51.47	10.83	.89
Readiness for discharge (RD)					
Parent’s status	61.46	13.56	64.27	10.78	.35
Child’s status	39.86	9.70	42.16	5.89	.48 (.04)^1^
Total score RD	231.57	46.71	239.72	29.66	.57
Presence per week at discharge (hours)	43.97	18.32	83.71	48.68	<.0001

*Notes:* Statistical tests of comparison**:** t-test for PSS: NICU, EPDS, NPST, and FCCQ. Wilcoxon test for PSS: NICU sights and sound, FCCQ respect, GSDS, BSES, RD. ^1^p value after controlling for maternal education

Mothers in the pod/SFR group reported significantly less overall NICU-stress (PSS: NICU Total score) compared to mothers in the former OW (Table 3). Examining the specific subscales of the PSS: NICU measure we found that the groups differed on two of the three subscales. OW mothers felt more restricted in their parental role (Table 3). For sights and sounds stress, the unadjusted mean difference approached but was not significant (*p* = .05). However, when controlling for maternal education the difference was significant. OW mothers reported significantly greater sights and sounds stress (adjusted Mean 2.79) compared to pod/SFR mothers (adjusted Mean = 2.45) (*p* = .008) (Table 3).

With respect to family-centered care, the groups differed significantly on one subscale: pod/SFR mothers reported greater respect from staff (Table 3). There was no significant group difference in mothers’ perceptions of the staff’s overall ability to provide family-centered care, however when controlling for maternal education there was a trend in favor of the pod/SFR group.

The two groups of mothers did not differ with respect to their total readiness for discharge, nor parent readiness (Table 3). However, controlling for maternal education pod/SFR mothers perceived their infant’s readiness for discharge to be greater than OW mothers (adjusted *M* = 42.2 versus 39.9).

Contrary to expectations, we found no significant differences between groups in depressive symptoms, nurse-parent support, sleep disturbances, and breastfeeding self-efficacy (Table 3). Almost half of mothers in both groups scored in the clinical range for depressive symptoms (score greater than or equal to 12 on the EPDS) (50.0% versus 45.0% in the OW and pod/SFR units respectively, χ^2^ = 0.51, *p* = .48). Moreover, most participants in both groups had sleep disturbances’ scores in the clinical range: 74.3% in the OW compared to 65.0% in the pod/SFR (χ^2^ = 2.13; *p* = .15). At discharge, the percentage of mothers reporting they were currently feeding at the breast was no different (65.7% in the OW versus 66.7% in the pod/SFR unit, (χ^2^ = 0.14; *p* = .71)).

For all mothers, we measured their presence at two points in time: at enrollment and again in the weeks proceeding the infant’s anticipated discharge. We found that at both times pod/SFR mothers were present significantly more hours per week than OW mothers, controlling for maternal education. At enrollment the adjusted means were 49 and 38 h per week respectively. Prior to discharge pod/SFR mothers were present on average 84 h per week compared to 44 h for the OW mothers (Table 3).

## Discussion

In contrast to many design studies mothers in our pod/SFR unit experienced two designs: their infant was admitted to a 6-bed pod and moved to a SFR for step-down care. Mothers completed questionnaires on their NICU-stress, depressive symptoms, family-centred care, support from nurses, and sleep in the early weeks after admission when their infant was in a pod. They reported significantly less overall NICU-stress compared to OW mothers, and the difference was significant after controlling for maternal education. It is noteworthy that mothers’ NICU-Stress Total mean scores and standard deviations for both our groups (OW *M* = 3.10, *SD =* 0.76) (pod/SFR *M* = 2.83, *SD* = 0.70) are very similar to those reported by Lester and colleagues in their comparison of an OW (*M* = 3.12, *SD* = .80) and a unit of all SFRs (*M* = 2.86, *SD* = .80) [11]. Thus, our finding suggests that 6-bed pods are less stressful for mothers than OWs and perhaps not much different from units of only SFRs.

The two PSS: NICU subscales scores on which our groups differed are those likely to be affected by design: sights and sounds and parental role restriction. In 6-bed pods, mothers may be less exposed to stressful sights and sounds due to the presence of fewer infants and staff in this smaller shared space compared to the former large OW. Our finding of lower parental role restriction stress for pod/SFR mothers when their infant was located in a pod is consistent with a study of a SFR unit [32] and extends this to a pod/SFR unit. Lower role restriction is of clinical importance as it is well documented that this is the most stressful aspect of the NICU stay for mothers [33, 34]. Mothers may experience less role restriction when there is adequate space for them to remain comfortably at their infant’s bedside. Moreover, staff may more readily involve them in their infant’s care as nurses indicate that in OWs chaos and lack of space and privacy are barriers to supporting parents [35, 36]. Our parallel study of staff nurses found no differences in nurses’ stress, work satisfaction, and ability to provide family-centered care between the OW and the pod/SFR unit.

Our study adds to existing evidence that design appears to influence maternal presence. We found at both enrollment and prior to discharge that pod/SFR mothers were present more than OW mothers (Table 3). In the weeks prior to their infant’s discharge, pod/SFR mothers were present twice as much as those in the OW (83.97 h per week versus 43.97). International surveys show that presence varies greatly across both units and countries [37]. Promoting parent presence is increasingly viewed as an essential component of NICU care given the benefits for infants long after discharge [11, 14, 15, 38], and current understanding concerning the neurobiology of parenting. In addition to differences in the hours of presence, the pattern may also differ with design. Lester and colleagues [11] found that mothers’ involvement (extracted following medical record review and including skin-to-skin contact, feeding, bathing, diapering and holding) was greater in SFRs immediately after birth, rapidly peaking in the first 2 weeks and sustained over time; while OW mothers’ involvement increased gradually. Unfortunately, we measured only presence, not involvement in infant care; and assessed it at only two points in time. There is currently no standard tool to measure presence, nor involvement, making comparisons across studies difficult; and both parent report and chart review data have limitations [5]. Future studies should measure both presence and diverse forms of maternal involvement throughout the hospital stay, and innovative approaches to measurement using new technologies are needed.

In the current investigation, pod/SFR mothers reported greater respect from staff than OW mothers. It should be noted that our participants completed this measure at enrollment while their infant was cared for in a 6-bed pod. This subscale of the family-centered care measure assesses privacy and the extent to which a parent feels welcome and “not like a visitor”. Qualitative work indicates that SFR mothers experience greater ownership over both their space and the care of their infant [39], and this may apply to small pods such as those at our study site. Parents identify having their presence welcomed by staff and amenities for them as factors that foster presence [40], suggesting that respect from staff and presence might be related.

The current study is the first to our knowledge to examine mothers’ readiness for discharge in units differing in design. In the weeks prior to expected discharge, pod/SFR mothers considered their infant’s readiness for discharge to be greater than their OW counterparts. Greater presence at the bedside may have provided an environment more closely approximating their home environment and helped them recognize that their infant was indeed ready to go home. In our qualitative study in the SFRs of this unit parents described how privacy allowed them to learn to care for their infant without feeling scrutinized [41]. Nurses have observed that SFR mothers “know more and do more” and in turn are more confident and prepared for discharge compared to OW mothers [39].

While a few previous design studies have examined mothers’ overall support, ours focused specifically on support from nurses using a reliable and valid measure. We found no difference between units. In contrast, an Australian survey reported that OW mothers had greater support from nurses than SFR mothers [39], and in-depth interviews revealed that in the SFR unit both mothers and nurses considered it more difficult to initiate interactions compared to in one large space where others were visible. Further examination of nurse support in units of differing design is warranted to better understand how to optimize support in different environments. Nonetheless our study indicates that in contrast to SFRs, mothers may feel equally supported by nurses in small pods as they do in OW units where nurses are always visible.

Similar to previous investigations [5, 32], we found no differences in mothers’ depressive symptoms. It may be that factors other than design play more of a role in depressive symptomatology among this at-risk group including previous history of depression [9], or studies may be under-powered to detect differences in rates. Pineda [5] observed that 20% of mothers had scores in clinical range on the same gold-standard measure of depressive symptoms administered in the current study. However, they utilized a cut-off score of 13 or more while we used a score of 12 and the proportion of women in the clinical range was higher.

Little is known as to how design may affect parents’ sleep when a newborn requires NICU care. Sleep disturbances at enrollment were common with more than 65% of women in each group having scores greater than 42. We did not explicitly inquire where mothers were sleeping, however their reports of their presence at enrollment showed that less than 15% of mothers in both groups were present after 11 pm, and there was no difference between groups. Given the lack of dedicated sleep spaces in both the former OW and the current pods, it seems likely that most mothers returned home to sleep. As with previous reports of parents’ sleep while a child is hospitalized, sleep, even when in a home or home-like environment, may be of reduced quality [42]. Factors other than design may affect maternal sleep such as the need to express breastmilk at night, worry about the child, and time spent commuting and caring for other family members [43].

## Conclusion

The current study is an important contribution to the evidence on NICU design as we assessed a design not often studied that combines pods and SFRs, with the 6-bed pods used for critical care and SFRs for step-down care. Mothers were less stressed in the pod-SFR unit compared to the OW, and their level of NICU-stress was similar to reports of mothers in an SFR unit. NICUs that include these two types of rooms used in this way may be optimal in some respects for parents. Early in the newborns’ hospitalization in a pod, mothers may feel supported and secure in the presence of nursing staff; while in a SFR for step-down care they may have the privacy needed at that time to consolidate their caregiving abilities in an environment approximating home prior to discharge.

The pre-post design is a study limitation; however, bias is lower in design studies conducted in the same hospital relative to studies comparing units at different hospitals where unit culture and care practices may differ. It should be noted that the design of the unit changed with the move to the new unit in a newly constructed wing, but the overall space increased greatly as well, and we cannot tease apart the effects of these simultaneous changes. Study strengths include the use of valid, reliable measures commonly used in studies of NICU parents and collecting data one-year post-occupancy so that staff and procedures could stabilize following the design transition. Further study of units combining small pods and SFRs exploring infant outcomes is indicated as our results show that compared to OWs these may be less stressful for parents, enhance family-centered care, as well as parent presence, important objectives in the care of fragile infants and their families. Future design studies should consider examining parent outcomes after discharge from the unit. Moreover, it would be important to include fathers to understand their presence, involvement and psychological well-being in units of differing designs.

## Data Availability

The datasets used and analyzed in this study are available from the corresponding author on reasonable request.

## Abbreviations

NICU: Neonatal Intensive Care Unit
OW: Open Ward
SFR: Single Family Room
